# Impact of the COVID-19 Pandemic on Cardiologists in a Country with Limited Resources

**DOI:** 10.5334/gh.878

**Published:** 2020-09-09

**Authors:** Muhammed Elhadi, Ahmed Alsoufi, Mohamed Abrahim Bin Zarti, Siraj Abulmida, Nafati Alnafati, Najwa Alfurjani, Ahmed Khaled, Munder Mansour, Ahmed Msherghi, Ahmed Tarek, Hazem Abdelkarem Faraj

**Affiliations:** 1 Faculty of Medicine, University of Tripoli, Tripoli, LY; 2 National Heart Institute, Tripoli, LY; 3 Tripoli University Hospital, Tripoli, LY

**Keywords:** COVID-19, SARS-CoV-2, cardiology, interventional, healthcare workers, PPE

**To the editor** 

The coronavirus disease 2019 (COVID-19) pandemic has disrupted healthcare systems in many institutions around the world [1]. It was originally thought that COVID-19 is mainly a respiratory disease. However, recent reports show that it affects the heart, causing myocarditis [2]. The COVID-19 pandemic caused high demands on clinical care systems, especially in critical care settings where there is an urgent need to treat and manage non-COVID-19 patients with co-morbidities, particularly cardiovascular diseases [3]. There is a high risk of neglecting patients with cardiovascular disease, especially in countries with limited resources that have been severely affected, and where many healthcare institutes have been unable to provide adequate care for their patients with cardiovascular disease [4]. Therefore, whenever possible, the impact of this pandemic should be minimized to ensure the continuity of healthcare services. In this study, we provide an overview of the status of cardiology departments during the COVID-19 pandemic in multiple centers in Libya, a country with limited healthcare resources.

A cross-sectional survey was conducted among cardiologists in four main tertiary centers in Libya, both online and on paper, between June 12 and June 29, 2020. Data regarding demographic characteristics, employment experience, personal protective equipment (PPE) availability, questions related to COVID-19 changes, and changes in catheterization laboratories and imaging status during the pandemic were recorded on anonymous data sheets. All participants provided informed consent before completing the survey.

During the two weeks of the study period, 118 completed survey responses were collected from four main cardiology departments with different units. Table 1 provides an overview of the status based on the respondents’ answers. Among the participants, 53 (44.9%) worked only in the government sector, 7 (5.9%) worked only in the private sector, while 58 (49.2%) worked in both. Among the respondents, 85 (72%) were cardiologists in training, while 17 (14.4%) were heads of units, and 16 (13.6%) were specialists or attendants. Regarding modifications during the COVID-19 pandemic, only 27 (22.9%) reported no changes in the working pattern in their cardiology units, while 50 (42.4%) reported reduced elective activity with interventions for urgent cases, and 41 (34.7%) reported cancellation of all elective activities with urgent intervention only.

**Table 1:** Basic characteristics of participants (n = 118).

Variables	Count or mean n = 118	Percentage

**Gender**		
Women	49	41.5
Men	69	58.5
**Age range**		
≤35	50	42.4
36–45	56	47.4
>45	12	10.2
**Participation in tele-medicine program**		
Yes	37	31.4
No	81	68.6
**Redeployment to other departments**		
Yes, to medical specialties	18	15.3
Yes, to emergency or intensive care units	20	16.9
No, still in cardiology department	80	67.8
**Working hours per week, (mean ± SD)**	47.17 ± 13.57	N/A
**Number of shifts per month, (mean ± SD)**	4.77 ± 1.33	N/A
**Changes during COVID-19 pandemic**		
Outpatient clinic cancelled	34	28.8
Changed the practice into triage	8	6.8
Video/telephone consultation	3	2.5
No changes	73	61.9
**catheterization lab status during COVID-19 pandemic**		
≤2 days per week	85	72.1
≥3 days per week	11	9.3
No cath lab procedures	22	18.6
**Cath lab imaging availability during COVID-19**		
Yes, available	92	78
No, or difficulty in availability	26	22
**Angioplasty and/or stenting program during COVID-19**		
No change, running as usual	16	13.6
Reduced	76	64.4
Stopped	20	16.9
Unavailable	6	5.1
**Acute coronary syndrome management changes in COVID-19**		
No change in management	87	73.7
Conservative management in non-ICU/CCU bed	17	14.4
Conservative management unless ruptured	14	11.9
**Availability of personal protective equipment (PPE)**		
Not available	20	16.9
Some are available	88	74.6
All are available	11	8.5
**Received training on using PPE**		
Yes	27	22.9
No	91	77.1
**Available of guideline/policy for managing COVID-19 patients**		
Yes	25	78.8
No	93	21.2
**Availability of COVID-19 testing**		
Antibody testing (fast kit)	2	1.7
Antigen testing (RT-PCR)	69	58.5
CT Chest only	47	39.8
**Number of patients waiting for cardiology procedures**		
<10	21	17.8
10–20	14	11.9
>20	83	70.3

Among the participants, 92 (78%) reported the use of a screening test such as computed topography (CT) or a COVID-19 test for patients suspected of having COVID-19, while 22 (18.6%) did not perform screening, and 4 (3.4%) reported screening all patients with cardiovascular disease. Regarding the testing abilities in cardiology units, 69 (58.5%) reported the availability of reverse transcription polymerase chain reaction (RT-PCR) testing, while 47 (39.8%) had only chest CT testing capability. Furthermore, 88 participants (74.6%) reported a unit policy for testing only healthcare workers (HCWs) suspected of having COVID-19, while only 8 (6.8%) reported testing for all HCWs, and 22 (18.6) reported the unavailability of testing for HCWs. Major reasons given for the low testing capacity were as follows: 46 (39%) noted a lack or shortage of equipment, while 26 (22%) reported lack of a hospital facility. Among the participants, 83 (70.3%) cardiologists reported that more than 20 patients waited for revascularization or any type of intervention due to delays caused by the COVID-19 pandemic.

Patients with cardiovascular disease are the most numerous patients with co-morbidities that are affected by COVID-19 [5]. There has been a rise in the number of cases that are waiting for procedures due to the shortage of supplies and redeployment of cardiologists in other departments. To our knowledge, this is the first study of the effects of the COVID-19 pandemic in a country with limited resources and restrictions on healthcare settings.

Healthcare systems in countries with limited resources are facing several challenges during the ongoing COVID-19 pandemic. As we approach the second wave, the challenges increase for fragile healthcare systems such as Libya’s. These hospitals have allocated their resources to fight the COVID-19 outbreak. Only 13.6% of respondents reported no changes in the stent insertion program while others reported delay or cancellation of this program. Over 70% of participants reported that more than 20 patients in their hospitals were awaiting cardiovascular procedures that were delayed or cancelled due to COVID-19 infection risk and disruption of healthcare services. This is shown by the fact that 70% of participants reported having less than two days allocated per week for catheterization laboratories. In addition, the PPE shortages and the disruption in interventional cardiology departments have decreased the ability of departments to manage these patients, as shown by the decreasing number of patients admitted and the cancellation of outpatient clinics and catheterization procedures. This puts more patients at risk of death or complications of their cardiovascular disease.

We acknowledge several limitations to this work. Indeed, it is intended merely as a preliminary view of a country with specific circumstances. Thus, we assume that future multi-national studies can confirm these findings in a broader way to give generalized results. There is also a need to assess the effects of delay in managing patients with cardiovascular disease, which increases the risk and the burden on healthcare systems, and to weigh the benefits and risks.

Our study was intended to raise awareness of cardiovascular patients affected by the disruption of healthcare services due to the COVID-19 pandemic. Our results emphasize the importance of caring for patients with cardiovascular disease and of allocating resources and efforts to manage these patients despite this disruption. There is a need to support countries with limited resource settings to help them cope with the challenges posed by COVID-19. Otherwise, the consequences could be catastrophic, given the fact that cardiovascular diseases are the most common cause of death worldwide and any disruption in cardiovascular healthcare services can increase the overall mortality and complications of these diseases [6].

## Data Accessibility Statement

The datasets used and/or analyzed during the current study are available from the corresponding author on reasonable request.

## Author Information

**Muhammed Elhadi:** Conceptualization, methodology, software, supervision, writing – original draft preparation. **Ahmed Alsoufi:** Data curation, writing – reviewing and editing. **Siraj Abulmida:** Data curation, writing – reviewing and editing. **Mohamed Abrahim Bin Zarti:** Data curation, writing – reviewing and editing. **Nafati Alnafati:** Data curation, writin – reviewing and editing. **Najwa Alfurjani:** Data curation, writing – reviewing and editing. **Ahmed Khaled:** Data curation, writing – reviewing and editing. **Munder Mansour:** Data curation, writing – reviewing and editing. **Ahmed Msherghi:** Data curation, writing – reviewing and editing. **Ahmed Tarek:** Data curation, writing – reviewing and editing. **Hazem Abdelkarem Faraj:** Data curation, methodology, writing – reviewing and editing.
